# Breath chemical markers of sexual arousal in humans

**DOI:** 10.1038/s41598-022-10325-6

**Published:** 2022-04-15

**Authors:** N. Wang, G. Pugliese, M. Carrito, C. Moura, P. Vasconcelos, N. Cera, M. Li, P. Nobre, J. R. Georgiadis, J. K. Schubert, J. Williams

**Affiliations:** 1 Max Planck Institute for Chemistry, Mainz, Germany; 2 University of Groningen, University Medical Center Groningen, Groningen, The Netherlands; 3 CPUP, Faculty of Psychology and Educational Sciences, University of Porto, Porto, Portugal; 4 Rostock University Medical Center, Rostock, Germany; 5 Climate and Atmosphere Research Center, The Cyprus Institute, Nicosia, Cyprus

**Keywords:** Biochemistry, Chemical biology, Biomarkers, Medical research

## Abstract

The chemical composition of exhaled breath was examined for volatile organic compound (VOC) indicators of sexual arousal in human beings. Participants (12-male, 12-female) were shown a randomized series of three emotion-inducing 10-min film clips interspersed with 3-min neutral film clips. The films caused different arousals: sports film (positive-nonsexual); horror film (negative-nonsexual); and erotic (sexual) that were monitored with physiological measurements including genital response and temperature. Simultaneously the breath was monitored for VOC and CO_2_. While some breath compounds (methanol and acetone) changed uniformly irrespective of the film order, several compounds did show significant arousal associated changes. For both genders CO_2_ and isoprene decreased in the sex clip. Some male individuals showed particularly strong increases of indole, phenol and cresol coincident with sexual arousal that decreased rapidly afterwards. These VOCs are degradation products of tyrosine and tryptophan, precursors for dopamine, noradrenalin, and serotonin, and therefore represent potential breath markers of sexual arousal.

## Introduction

When a human being encounters a stimulus of some kind, under particular conditions, a physiological change can occur from a generally quiescent state to one of arousal. Such arousing stimuli may take many forms; fear from impending danger, euphoria associated with success, or desire for a sexually attractive person. The trigger for arousal may be via sight, sound, smell, or touch but the result is a change in the metabolic and physiological processes within the body.

Arousal is perhaps the most important brain state in humans. Without it no overt behaviors, cognitive processes, and emotions are possible, and without its proper function, responses are out of tune with the needs imposed by the context. At the onset of arousal, groups of neurons with wide ascending and descending projections, use glutamate, noradrenaline, dopamine, and hypocretin as neurotransmitters to enable fast and robust upregulation of the entire neuroaxis^1^. In the body, arousal manifests as increased muscle activity—both those associated with movement and of those that make our viscera, glands and blood vessels function in line with the increased demands.

This ‘generalized’ type of arousal must somehow interact with the more specific arousal types that turn on distinct functions in the body and brain. A prime example of this is sex. The motor expression typical of sex, that is distinct from other behaviors, is arterial dilatation in specialized vascular tissue in the genital area, leading to enhanced genital blood flow and vasocongestion, which in turn produces penile and clitoral tumescence, and vaginal lubrication (known as ‘physiological sexual arousal’). These externally visible changes are driven by peripheral nerves that activate vasoactive compounds (mainly nitric oxide and vasoactive intestinal polypeptide) in the muscle wall of genital arteries^2–4^. At the same time, sexual arousal also involves generalized arousal in the form of increased motor behavior, as well as pupil dilatation, increased sweating, respiration, and cardiovascular activity^5,6^. These responses are largely based on the stimulation of noradrenergic nerve fibers. At the brain level too, the perception of arousal as sexual and pleasurable occurs together with the perception of generalized arousal and associated changes in cognitive processes (known as subjective sexual arousal). Sex steroid hormones (oestradiol and testosterone) produced by the gonads in response to brain signals in turn modulate the responsivity of genital vascular tissues and of specific brain areas, thereby acting to steer body and brain to sexual activity and to amplify sexual behavior when it occurs^7,8^.

Clinically, issues that relate to sexual arousal like sexual dysfunction and sexual transgressive behaviors, are not only very prevalent^9,10^ but are also significantly associated with mental health^11^ and ultimately contribute to decreased quality of life^12^. It is therefore important to be able to assess sexual arousal. Yet, existing assessments are suboptimal because they are prone to subjectivity bias (self-reports, surveys), are unspecific (e.g. pupil dilation), or are invasive/intrusive (genital measurements), the latter even more so in women than in men^13^. Recent developments include the use of thermography to assess genital blood flow. However, despite being less invasive, this technique still involves some degree of intrusiveness (recording thermal imaging of the genitals)^14^ Moreover, none of the existing measurements are able to reliably compare the sexual arousal of men and women. This limits our understanding of the human sexual arousal response.

Recently it has been shown that emotional responses of cinema audiences can be tracked through high frequency measurements of chemical compounds in the air^15^. These trace gas phase compounds are emitted by people through the breath as they react to moments in the film. Several hundred volatile organic compounds were monitored at parts per billion levels or lower in air that had entered the cinema through under-seat vents, swept over the audience, and exited though ceiling vents in the screening room^16^. Variations in isoprene, a breath borne compound that is stored in muscle tissue and released on breath with muscle movement, was shown to correlate the age classification of films^16^. This opens the possibility that exhaled VOCs could be used to monitor physiological changes in the body during the sexual arousal response in a completely non-invasive fashion.

In this study we have continuously analyzed over one hundred volatile organic compounds in the breath of 24 individuals (12 male, 12 female) as they watched sections of a nature travel documentary (neutral quiescent conditions), a horror film (anxiety condition—non-sexual), a football match (positive arousal—non-sexual) and an erotic film (sexual condition). In addition to the novel breath analysis of volatile organic compounds, carbon dioxide (CO_2_), and all standard measurements of sexual arousal were made in parallel. These included a penile gauge or vaginal photoplethysmograph and thermal imagery of the genitals. This dataset therefore allows us to test whether sexual arousal, as determined by the standard devices, can be perceived with specific chemicals that are generated within the body as part of the sexual response and vented on the breath.

## Results

### Overview of breath compounds

Among 110 m/z detected from the exhaled air by proton transfer time of flight mass spectrometer (PTR-ToF-MS), only 21 of them showed higher exhaled levels than the inhaled (room air) levels. These 21 exhaled VOCs were included for further analysis in this study. Chemical identification assignment and statistical information including the mean, median, maximum, minimum and standard deviation of these selected exhaled VOCs from all the participants during each film clip are listed in Table S1. Methanol, acetone and isoprene were the most abundant VOCs detected, with the mixing ratios ranging from 176 to 1528 ppb for methanol, 110 to 561 ppb for acetone and 29 to 384 ppb for isoprene over the experimental period. The dominance of these three compounds in human breath is consistent with many previous studies^17–19^. The main focus of this study was the relative variation of VOC breath markers in response to the different film clips. Potential breath-gas arousal markers were assessed with reference to the temporal correlation with the standard physiological measurements.

Based on the physiological parameters the participants were not all aroused to the same extent, with strong and weak genital responses present in the dataset. Here we assess the whole dataset in general terms, before focusing on individuals (separated by gender) to assess specific chemical markers.

**Figure 1:**
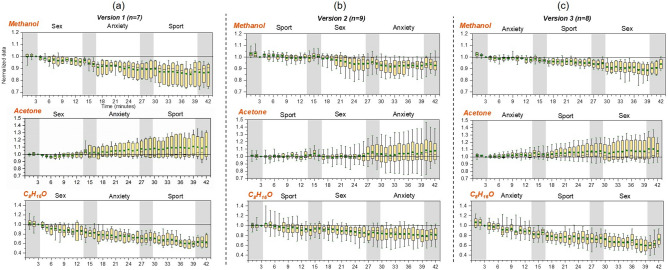
Time series of methanol, acetone and C_8_H_16_O (normalized to the 3rd minute of the first neutral clip based on mixing ratio data) under three film clip playing orders: (**a**) version 1 (sex–anxiety–sport) including 7 participants (male: 4, female: 3); (**b**) version 2 (sport–sex–anxiety) including 9 participants (male: 5, female: 4); version (**c**) (anxiety–sport–sex) including 8 participants (male: 3, female: 5). Shaded area represent the time when neural clips were played. The boxes represent 25% to 75% of the dataset, with the circle and central line indicting the mean and median values, respectively. The whiskers indicate the minimum and maximum data points. The horizontal solid line in each sub-plot represents the value at 1.0 as a reference.

Exhaled concentrations of methanol, acetone and a compound with molecular formula of C_8_H_16_O changed consistently and uniformly along the experimental timeline regardless of the film clip order (Fig. 1), indicating that breath emissions of these compounds were time-dependent rather than emotional stimuli dependent. Methanol, acetone and C_8_H_16_O are therefore not suitable as breath-borne markers of arousal, but rather are products of underlying continuous metabolic processes. Compared to the third minute of the first neutral clip, the mean level of methanol and C_8_H_16_O decreased by 10% and 32%, respectively over the experimental time while the mean level of acetone increased by 9%. For methanol and acetone, data in the range of 25–75% (boxes in Fig. 1) became more widely distributed along with the time, meaning that the variation within participants became larger towards the end of the whole experiment. Other breath-borne VOCs did not show time-dependent trends, indicating that breath concentration of these VOCs might be related to emotional stimuli.

### Sexual arousal vs. film clips

The results obtained indicated that experimental manipulation overall was successful. The sexual stimuli elicited both genital and subjective sexual responses compared to non-sexual stimuli (p < 0.0001, Table S2). Subjective sexual response to the sexual clip was moderate to high in both women (*M*_*Female*_ = 5.50, *SD*_*Female*_ = 1.51) and men (*M*_*Male*_ = 7.20, *SD*_*Male*_ = 1.03) although significantly higher for the latter. Interestingly, female participants reported significantly higher subjective sexual arousal after watching the sport clip (*M* = 1.50, *SD* = 0.47) than the anxiety clip (*M* = 0.08, *SD* = 0.08), p < 0.05), while female genital response has no significant difference between sport and anxiety clip. Subjective arousal after watching the sports film was nonetheless lower than subjective arousal reported after the sexual film, p < 0.0001. A good correlation between the subjective arousal response and genital response was only found among male participants during the sex clip with R^2^ = 0.72 (p < 0.05, see Fig. S2).

**Figure 2:**
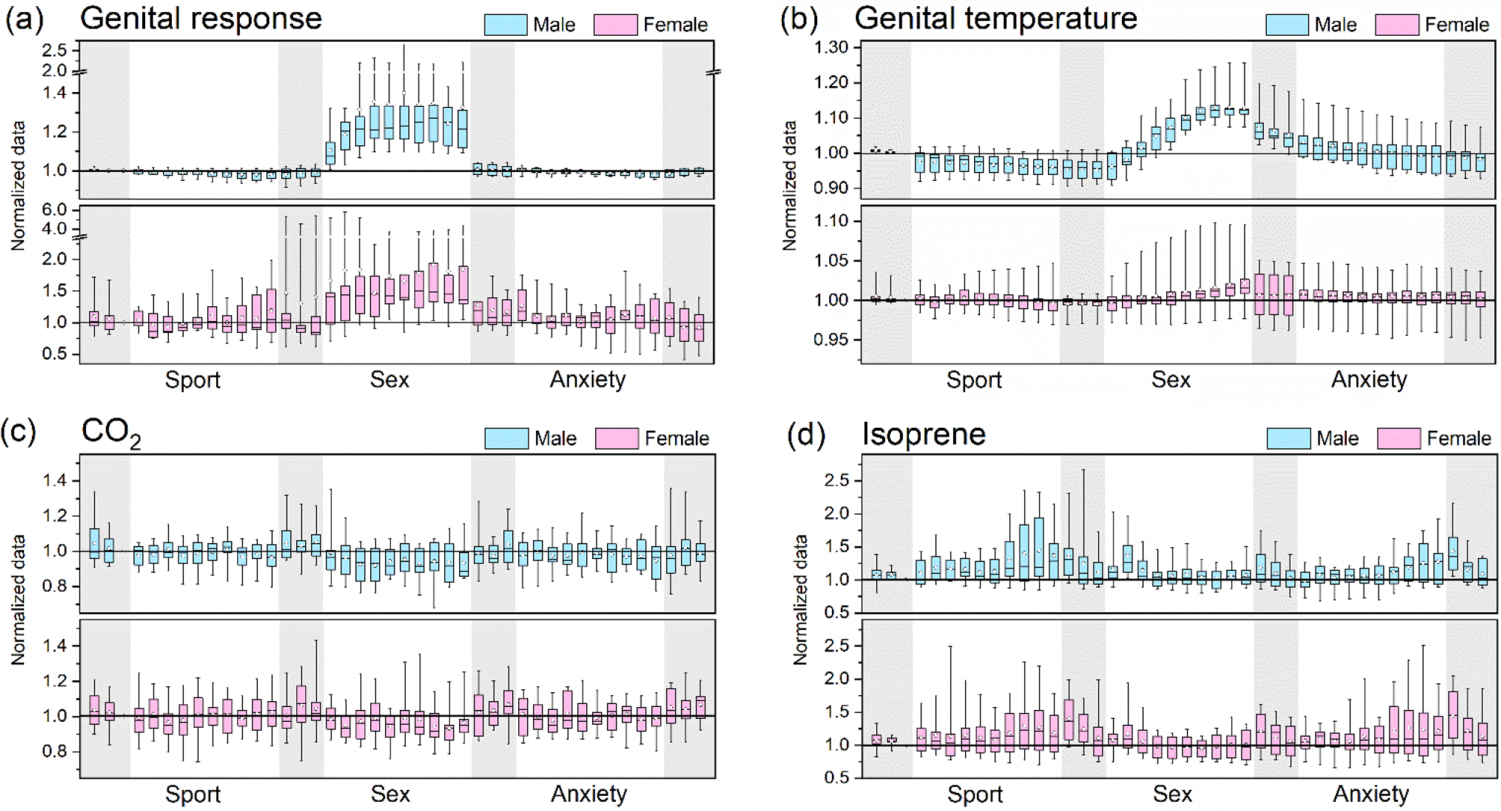
(**a**) genital response (male: penile circumference; female: vaginal pulse amplitude) (**b**) genital temperature (male: glans of the penis; female: clitoris) (**c**) exhaled breath CO_2_ and (**d**) isoprene in breath for male (in blue) and female (in pink) participants during each minute of different film clips. Data were normalized to the 3rd minute of the first neutral clip based on measured raw signals. Shaded area represents the time when neural clips were played. The boxes represent 25% to 75% of the dataset, with the circle and central line indicting the mean and median values, respectively. The whiskers indicate the minimum and maximum data points. The horizontal solid line in each sub-plot represents the value at 1.0 as a reference.

**Figure 3:**
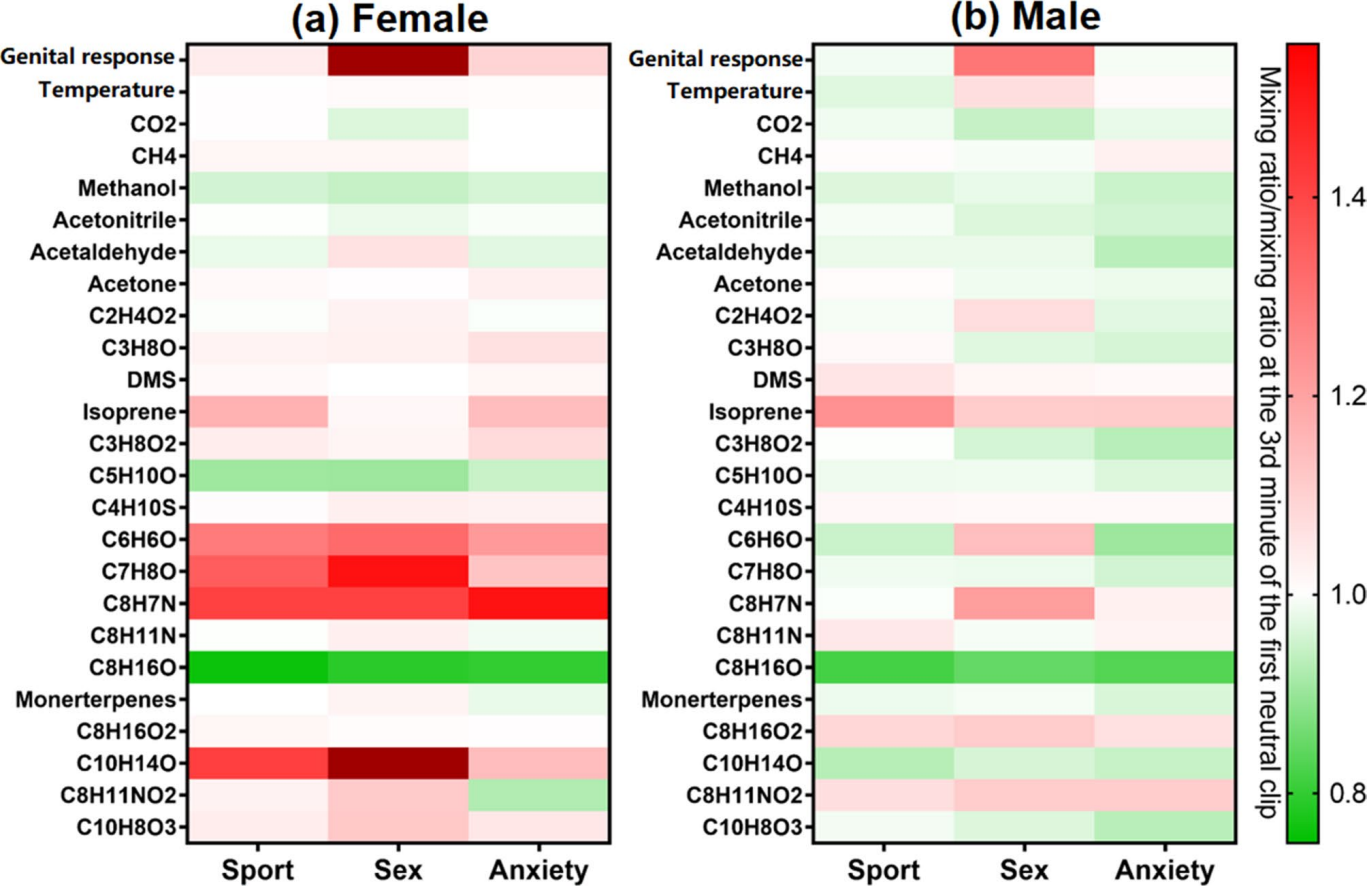
Mean levels of normalized genital response (male: penile circumference; female: vaginal pulse amplitude), temperature (male: glans of the penis; female: clitoris) and selected VOCs in exhalation of (**a**) female participants and (**b**) male participants during each film clip.

Figure 2a,b show the minute-by-minute data distribution of the normalized genital response, as assessed by penile circumference and vaginal pulse amplitude, and the normalized genital temperature. For genital temperature, the ROI 1 (glans of the penis for males and clitoris for females, see Fig. S1) was selected for this purpose because the thermal signals from those regions were the most responsive during the sex clip. Visual inspection of the genital response of men and women revealed greater similarity in response compared to their genital temperature variations. In general, male participants showed a higher and more distinct relative increase of genital response and genital temperature during the sex clip than female participants. By looking at normalized mean levels during each film (Fig. 3), the genital response was significantly elevated for participants during the sex film clip (p < 0.0001, Table S2), indicating that the sexual stimulation was effective on a physiological level in both gender groups. However, in terms of the genital temperature (for the glans of penis and clitoris at least), for male participants the mean temperature was significantly higher during the sex clip than during the sport and anxiety clips with p < 0.0001 (Fig. 3b), while for female participants no such pattern was observed (Fig. 3a). In women, the genital temperature during the sex clip was only significantly higher than the temperature during the sport clip (Table S2).

### Exhaled breath VOCs vs. film clips

Exhaled breath VOCs showed variations in concentration associated with the different clips, even though the relative change was less distinguishable compared to the genital response (Fig. 3). For female participants, breath levels of CO_2_ and isoprene during the sex clip were significantly lower compared to the anxiety and sport clips (p < 0.05, Table S2). For male participants, CO_2_, C_2_H_4_O_2_ and C_6_H_6_O were found to have a significantly lower, higher and higher breath level, respectively, for the sex clip comparing to the other two clips (p < 0.05, Table S2). The relative change of CO_2_ during the sex clip was in general only 3–4% lower than that during the anxiety and sport clips but was significant for both genders (p < 0.05). The minute-by-minute box plot (Fig. 2c) also shows that breath CO_2_ appears to lower slightly in concentration for both genders during the sex clip. Isoprene was significantly decreased during the sex clip compared to the sport clip for both genders (males:13%, females:15%, p ≤ 0.001). For women during the sex clip, the isoprene also had a significantly lower level compared to the anxiety clip (12%, p < 0.01). For the 1-min data distribution shown in Fig. 2d, participants showed not only elevated isoprene concentration during the sport and anxiety clips but also larger variations among each other reflected by the length of the box representing 25–75% data distribution. Interestingly, isoprene level peaked at the beginning of the sex clip (the second minute) for both genders.

From Fig. 3, it can be seen that several other measured VOCs, C_10_H_14_O, C_7_H_8_O, C_8_H_11_NO_2_ and acetaldehyde for female participants, and C_8_H_7_N for male participants showed large relative changes during the sex clip compared to the anxiety clip and the sport clip. However, no significant difference was identified between the sex clip and the other two clips (Table S2) for these VOCs, indicating the mean values were largely affected by outliers. In such cases, the VOCs identified as having a distinguishable change during the sex clip might be specific to an individual rather than being representative of the whole group of participants. Among the female participants, one subject (No. 39) had substantial elevation of C_10_H_14_O, C_7_H_8_O, C_8_H_11_NO_2_ in her breath starting in the end of the sport clip until the end of the sex clip, which caused a significant deviation in terms of the mean values. While for acetaldehyde, subject No. 20 had a much higher relative increase compared to the first neutral clip in her breath than other participants, affecting the mean values. For male participants, two persons with a strong physiological response (No. 6 and 10) had substantial elevated breath levels of C_8_H_7_N, C_6_H_6_O and C_7_H_8_O during the sex clip.

### Potential markers for sexual arousal

Among all participants, several VOCs showed change according to the genital response of certain individual participants (1 female and 2 male participants). Although the data from those participants were considered as outliers in the previous section, as there were no experimental errors identified and the breath-genital related change occurred in different clip playing order, it is unlikely that those outliers coincidentally followed the genital response pattern. Furthermore, genital response and genital temperature data was rated highly in terms of quality for those individuals. Therefore, we may use these individuals to characterize the breath marker responses for each gender in real time.

#### Female

**Figure 4:**
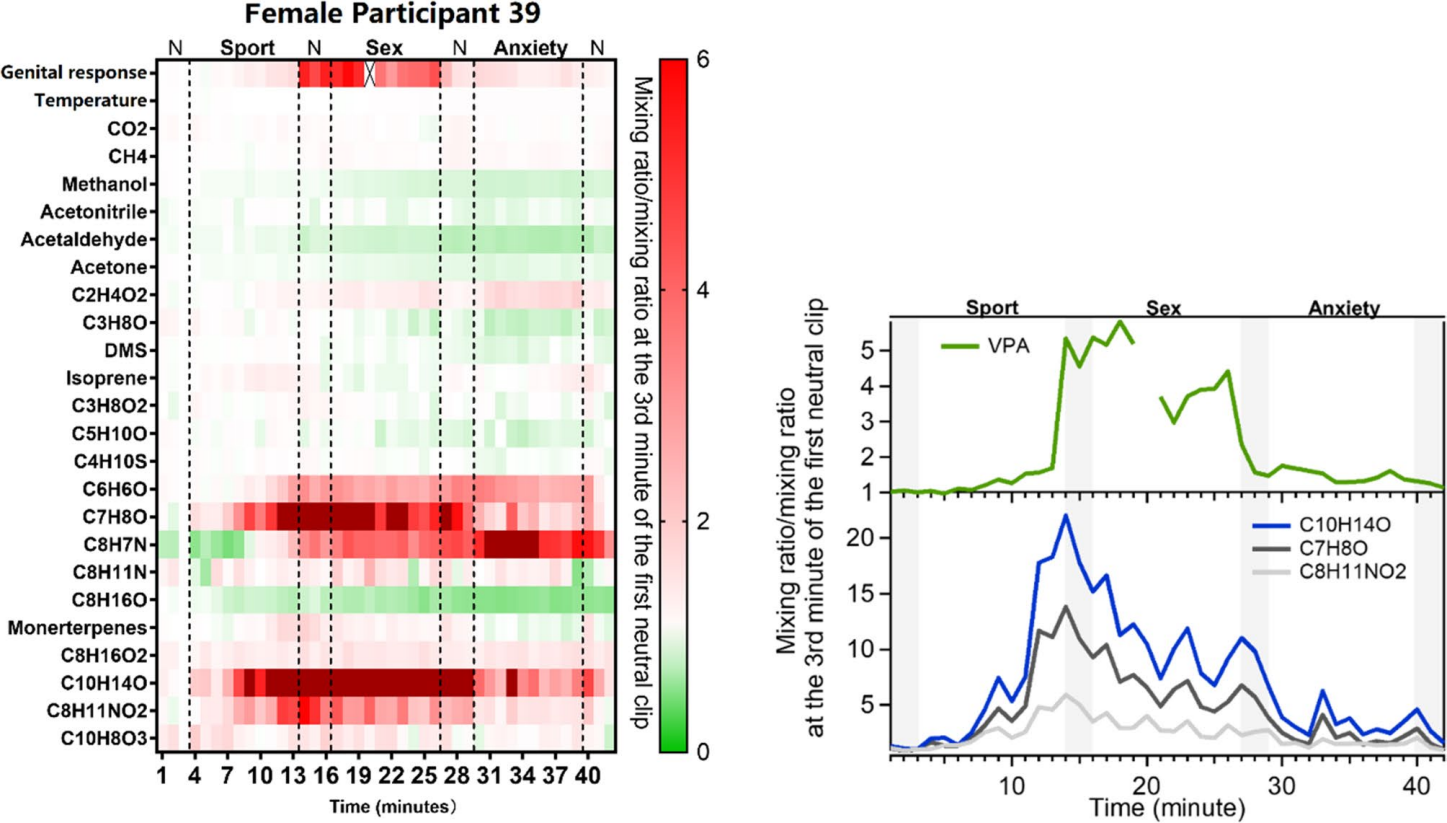
Heat plot of breath compounds together with genital response (vaginal pulse amplitude, VPA) and temperature (clitoris) of female volunteer No. 39 over the experimental time (upper figure), and time series of selected VOCs having large variations (lower figure). The cross in the heat plot indicates data over that minute was not available. Shaded area on the right plot represents the time when neutral clips were played.

Figure 4 shows the time series of all breath compounds together with the genital response and genital temperature for female volunteer No. 39. Interestingly, the genital signal started to rise slightly in the middle of the sport clip and increased substantially during the post-sport neutral clip and the sex clip, peaking at the second minute of the sex clip. Levels of three VOCs in breath (C_10_H_14_O, C_7_H_8_O, C_8_H_11_NO_2_) were elevated nearly at the same time, peaking at the first minute of the post-sport neutral clip, which was 3 min earlier than the peaking time of genital response. The levels of genital response and three breath compounds were somewhat lower during the second half of the sex clip. C_6_H_6_O and C_8_H_7_N started to increase at the same time during the sport clip but kept high till the end of the last film clip (anxiety). The time series of the above-mentioned VOCs for all female participants are shown in Fig. S3.

#### Male

**Figure 5:**
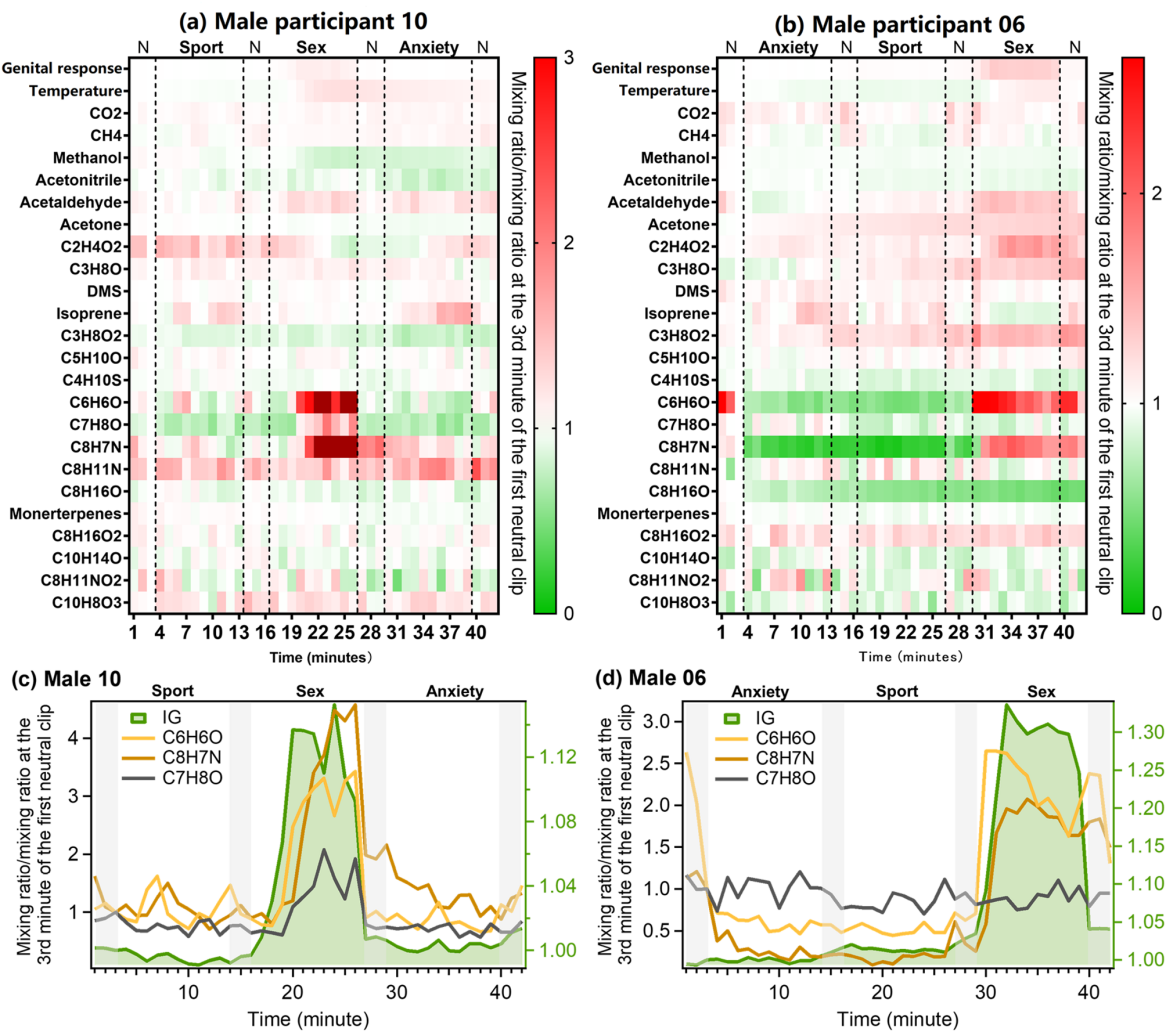
Heat plot of breath compounds together with genital response (penile circumference, IG) and temperature (glans of the penis) of (**a**) male volunteer No. 10 and (**b**) male volunteer No. 06 over the experimental time and time series of selected VOCs having large variations for (**c**) male volunteer No. 10 and (**d**) male volunteer No. 06. Shaded area in (**c**) and (**d**) represents the time when neural clips were played.

Two male participants (No. 6 and No. 10) that according to the physiological genital data responded strongly to the film clip stimulus, were found to have significant elevation for C_6_H_6_O and C_8_H_7_N during the sex clip with simultaneous increase of genital response, despite the film clip playing order being different for those two participants (Fig. 5). C_7_H_8_O, a VOCs that increased with genital response for female subject No. 39, also showed an increase for male subject No. 10 and was associated with the genital response during the sex clip (Fig. 5a,c). No such increase was identified for male subject No. 6 (Fig. 5b,d). The time series of the above-mentioned VOCs for all male participants are shown in Fig. S4.

## Discussion

The results presented above show that the overall experimental procedure was successful. The films elicited various degrees of arousal response in the participants that could be monitored with physiological and subjective measurements, and compared with simultaneously measured breath-borne volatile organic compounds. Genital response and genital temperature, indicative of increased genital blood flow, proved to be good indicators of sexual arousal, although all are invasive measurements requiring particular conditions of measurement. In men, the correspondence between genital response and subjective arousal was much stronger than in women (where it was virtually absent). The latter is consistent with the frequently reported discordance of subjective and genital arousal in women^20^.

From the breath-borne VOCs measured, some showed a consistent trend throughout each experiment irrespective of the order of the films. Acetone, methanol and C_8_H_16_O tentatively identified as octenol showed this time-dependent bias in their time series, indicating that their emission is independent of arousal or type of emotional stimulation and associated with continuing metabolic processes. The major source of exhaled acetone is decarboxylation of acetoacetate which in turn may arise from lipolysis, glycolysis^21^. The steady increase of breath acetone concentrations until the end of the study can be attributed to increased lipolysis due to the fasting effect, namely that the participants were not eating during the experiment^22^. Foods such as fruits, vegetables, meat and drinks, and gut microbiota activity are the likely sources of methanol and octenol in the healthy human body^19,23,24^. Previous studies have shown that the speed and amount of alcohols absorbed after ingestion are largely dependent on the rate of gastric emptying^25,26^. The steady decrease of exhaled concentrations of methanol and octenol may therefore be explained with the continuous gastric emptying due to subject not eating during the experiment.

Exhaled breath concentrations of isoprene and CO_2_ were significantly lower on average for both genders during the sex film clip compared to the other clips. Both isoprene and CO_2_ breath concentrations strongly depend on the ratio of alveolar ventilation to blood flow through the alveoli^27,28^. Thus, the lower exhaled CO_2_ and isoprene concentrations during the sex clip might be related to a blood flow dynamic in the genital region that ultimately affect perfusion of alveoli and other muscular regions. Specifically, genital arousal is characterized by an initial phase of increased genital blood flow and a second phase where the blood rather pools in the genital vascular tissue causing genital engorgement^29^. In our data, isoprene showed a peak at the beginning of the sex clip for both male and female participants. Previous studies have suggested that skeletal muscles may act as extrahepatic production and storage sites of substantial amounts of isoprene in the body^30–32^. We infer that the relative higher isoprene breath concentration at the second minute of the sex clip is due to increased genital blood flow to the genital area that purges-out the isoprene from the genitopelvic muscle compartment. The following established genital engorgement may explain the steady decrease of exhaled isoprene as well as CO_2_ levels during the remaining minutes of sex clip.

Several studies showed that isoprene concentrations in breath are considerably more sensitive, compared to CO_2_ concentrations, to changes in alveolar ventilation-perfusion ratio induced by changes in body positions and physiological maneuvers, which is mainly due to the lower solubility of isoprene compared to CO_2_^33,34^. Therefore, we may also consider the possibility that the low inter-individual variation of exhaled isoprene during the sex clip is likely due to the participants being more concentrated and moving less compared to the other clips. This interpretation is consistent with findings that sexual stimuli attract attention more than other emotional stimuli^35,36^. In addition, recent findings using eye-tracking measures indicated that individuals tend to have fewer but longer eye fixations during exposure to sexual films compared to neutral clips^37,38^, suggesting a stronger attentional focus to erotic cues that may be related to cognitive processing of sexual stimuli. Therefore, although breath isoprene can be a potentially useful marker of sexual arousal, caution is advised as it is also strongly dependent on hemodynamic and respiratory parameters such as cardiac output and pulmonary ventilation which in turn are highly variable depending on several factors (e.g. changes in body position).

In search of more specific candidates for breath-borne markers of genital sexual responding, we analyzed participants individually to see if there were VOCs that changes markedly and specifically during the sexual film clip, even when the films are shown in a different order. Male participants No. 6 and No. 10 showed clear increases in compounds with masses C_6_H_6_O and C_8_H_7_N, uniquely during the sexual film clip and in proportion to the degree of measured physiological arousal. Furthermore, these compounds decreased rapidly after the stimulus ceased. Male participant No. 10 and female participant No. 39 had obvious increases in the compound with mass equivalent to C_7_H_8_O. These aforementioned three masses were tentatively attributed to phenol (C_6_H_6_O), cresol (C_7_H_8_O) and indole (C_8_H_7_N) which are produced by the gut microbiota from tryptophan and tyrosine, respectively^39,40^. Increased breath concentrations of indole and phenol were observed after the ingestion of a protein oral challenge as result of gut bacterial degradation of tryptophan and tyrosine^41^. Tryptophan and tyrosine are the precursors of serotonin, dopamine, and noradrenalin, neurotransmitters which are rapidly generated upon sexual arousal and play a critical role in human sexual behavior^7,42,43^. Dopamine and serotonin have functionally opposing roles in behavior, whereby dopamine is associated mainly with connecting reward and action and serotonin with connecting punishment and withdrawal^44^. Yet, behavior requires both of these forces. Even when sexually aroused, behavioral control may be required. The present experiment may have demanded a considerable amount of behavioral control on the part of the participants, which they were able to exert despite being aroused (as the genital data show). Therefore, the masses we identify as providing the potential for sexual arousal breath markers are likely to be linked to known chemical mechanisms associated with arousal occurring in the body. A possible explanation of these observations is that in response to the sexual stimuli, the body levels of available tryptophan and tyrosine for neurotransmitter biosynthesis increase, leading in turn to an increase in levels of their metabolites such as phenol, cresol and indole on breath. Despite this connection, the internal physiology and in particular the high speed of the onset and end of the response is not fully understood.

Phenol, cresol and indole in exhaled breath have been reported in several previous studies, while none of them focused on breath VOC variations in participants under emotional stimuli^45–50^. Phenol, cresol and indole were not observed in the emotion tracking cinema study^16^. This is likely because none of the films screened in that study contained sustained sexually arousing content. Interestingly, while the cinema study noted higher isoprene variability and CO_2_ emission when audiences watched horror-type films with higher age classifications^16^, this study showed the opposite response during sexual arousal.

In the case of female participant No. 39, we also detect VOCs associated with the time of sexual arousal. These were C_7_H_8_O, C_8_H_11_NO_2_, C_10_H_14_O, and which correspond to the compounds of cresol, dopamine, and the tentatively identified 2-Ethyl-4,5-dimethylphenol, respectively. The presence of dopamine, detected in the breath again provides a direct link to the arousal response, although this compound was not markedly elevated in those two male participants (No. 6 and No. 10). Interestingly in the case of female participant no. 39, the physiological arousal and concomitant breath marker emission began shortly before beginning of the sexual film, at the end of the sport film clip. This may represent a gender biased limitation of the experiment. At the end of the sports film clip, which was intended to be a non-sexual positive arousal, several of the triumphant male football players. For heterosexual males this would indeed be interpreted as intended, as a positive non-sexual stimulus. However, it can be that female participant 39, already primed with the knowledge of conducting a sexual research experiment, became aroused when exposed to the celebrating of athletes. Meanwhile, previous studies report that women are more prone to respond with sexual arousal to a wider range of stimuli, while men show a much more specific response (to preferred sex stimuli)^51^. Indeed, in our study, women reported significant higher subjective sexual arousal for the sport clip compared to men. In addition, women reported significantly higher subjective sexual arousal to the sport film compared to the anxiety film. An alternative explanation for this increase starting just prior to the sexual stimulation could be dopamine’s role in anticipating the delivery of rewarding stimuli^52^, including sexual stimulation^7^. In future studies a more gender-neutral film should be chosen for the positive non-sexual arousal stimulus.

In conclusion we have identified several compounds that appear in the breath of humans experiencing sexual arousal. Based on the results presented here phenol and indole appear to be good potential indicators of sexual arousal on breath in males, being rapidly generated and removed so that their emission is in close synchrony with the sexual stimulus. Although less well associated with the timing of the sexual film in this study, the female physiological arousal was also linked to specific breath marker compounds, some of which are directly related to arousal (e.g. dopamine). Due to the small sample of tested participants in this pilot study, potential sexual-arousal related compounds were only detected in limited participants and the gender dependency for those potential compounds is still unclear. Therefore, it is pertinent to question how generalizable these effects are in light of the responses presented. It should be noted that previous experiments of this kind using participants but without chemical sensing have also shown a spectrum of response strengths from none to strong, so a limited number of strong responders was expected. Among these cases examples of clear, rapid, chemical breath chemical changes were detected that could be related to known neurochemical control processes within the body. However, this opens the possibility of being able to detect human sexual arousal non-invasively on breath, which has a large number of important applications. In the field of sexual research current methods involving attachment of sensors to genital regions imposes restrictions on the number of participants and time required for an assessment. Being able to gauge human sexual response on breath offers this research area the possibility to investigate responses under more natural conditions. Clinically, it could potentially contribute to less invasive methods to diagnose or verify sexual dysfunctions and paraphilic disorders. Based on the success of this study, a largescale experiment is clearly warranted so that the responses revealed in this first study can be characterized over a larger set of individuals, with additional detectors, and under different conditions. It is interesting to reflect that when sexually aroused both male and female participants began to broadcast specific chemical signals on their breath that were directly related to their arousal state. Whether such signals are emitted and then perceived by others during such activities as talking, kissing, or hugging is a fascinating open question that should be a focus of future studies. Further study of monitoring exhaled VOCs in parallel with VOCs emitted by skin or sweat and markers of brain neuromodulator levels would provide more information to better understand the link between entire human volatilome and sexual arousal.

## Methods

### Participants

A total of 24 individuals participated in the study (12 women and 12 men) (mean age = 26.7 years, SD = 5.03). Most participants had an academic degree (79.2%), were in a stable relationship (70.8%), and were sexually active. See Table 1 for additional demographic information. Participants were recruited using social media. Inclusion criteria were: being older than 18 years, heterosexual orientation, absence of psychopathology (e.g., depression, anxiety), medical problems, sexual dysfunction, or use of medications that may interfere with sexual function. The inclusion criteria were assessed using validated measures of psychopathology, by means of Beck Depression Inventory (BDI)^53^ and State Trait Anxiety Inventory (STAI)^54^, sexual dysfunction, by means of International Index of Erectile Function (IIEF)^55^ or the Female Sexual Function Index (FSFI)^56^ and a checklist of medical conditions and medications. The study was performed in accordance with the Declaration of Helsinki and was approved by the Ethics Committee of the Faculty of Psychology and Educational Sciences of the University of Porto. Informed consent was obtained from all participants before the study.

**Table 1:** Sociodemographic characteristics of participants (n = 24).

	Men (*n* = 12)	Women (*n* = 12)
Age, mean ± SD (min–max)	28.8 ± 4.91 (19–37)	24.5 ± 4.30 (19–32)
**Education level, *n* (%)**		
10–12 years	3 (25.0%)	2 (16.7%)
College degree	9 (75.0%)	10 (83.3%)
**Relationship status, *n* (%)**		
Single	3 (25.0%)	3 (25.0%)
Committed relationship	9 (75.0%)	8 (66.7%)
Multiple partners	–	1 (8.3%)
Had children, *n* (%)	1 (8.3%)	–
Smoker, *n* (%)	2 (16.7%)	2 (16.7%)

### Stimuli

Arousal was induced by showing the participants three 10-min film clips specific to type of arousal: (1) sexual, (2) anxiety, (3) positive non-sexual. This way we could study breath emissions in relation to sexual arousal while canceling out possible effects of generalized arousal and valence. The film clips were played in a randomized order and with a 3-min neutral film clip played in between each film to re-establish baseline conditions. The sexual film displayed a heterosexual couple engaging in oral and penile–vaginal intercourse. The film was chosen from a database of the Portuguese SexLab based on the high levels of sexual arousal elicited in men and women. The film was successfully used in previous research to induce sexual arousal^37^ and was primarily selected based on the findings of a pilot study where a sample of men and women rated the most sexually arousing clips from a total of 10. The anxiety-inducing film was a 10-min clip from the Russian roulette scene of The Deer Hunter (Cimino, 1978). This clip was chosen for its capacity to induce sustained anxiety over a relatively long period of time. “The Deer Hunter” depicted captives forced to play Russian roulette and was successfully used by Gomez et al*.* to induce an anxious mood state^57^. The sports film was a 10-min clip consisting of footage from the UEFA Euro Championship 2016 final, and it was chosen as a “positive non-sexual stimulus” as it depicted Portugal winning the UEFA Euro cup and players celebrating, which was expected to elicit positive emotions in the Portuguese participant. Sport clips were successfully used in previous studies to act as the non-sexual arousal control^58,59^. Finally, the neutral videos were three 3-min clips taken from a travels documentary with voiceover narration removed and replaced with instrumental music. These clips were used for their neutral emotional valence, and their non-arousing properties.

### Measures of genital arousal

Genital arousal was measured using several methods that are used in sexual research and physical examination in the context of sexual problems: vaginal pulse amplitude (vaginal blood flow), penile circumference (penile blood flow), genital temperature (blood flow to superficial genital structures).

#### Vaginal pulse amplitude

Genital arousal in women was measured using a vaginal photoplethysmograph^60^. The AC signal was taken as a measure of vaginal pulse amplitude (VPA). The Biopac MP 100 system (BIOPAC System Inc.) with PPG 100 amplifier, and AcqKnowledge 4.0 software was used for data acquisition and processing. VPA was sampled at 125 Hz and low-pass filtered (5 Hz). VPA signals were visually inspected and movement artifacts were removed, after which peak-to-peak amplitudes were calculated.

#### Penile circumference

Genital arousal in men was measured using an Indium-Gallium strain gauge (IG)^61^. Penile circumference was recorded continuously during baseline and stimulus conditions. The Biopac MP 100 system (BIOPAC System Inc., Goleta, CA, USA) and DA100 amplifier with AcqKnowledge 4.0 software was used for data acquisition and processing. Penile circumference signal was sampled at 1000 Hz and low-pass filtered (10 Hz). Genital responses were defined in terms of differences between sexual and baseline stimuli.

#### Genital temperature

Genital temperature data in men and women were collected using thermographic camera FLIR E60 (Wilsonville, OR, USA) placed on a tripod, with sensor focal array size of 320 × 240, NEDT (noise equivalent differential temperature) < 50 mK. Emissivity was set at 0.98. The sampling interval was set at 12 frames per minute (one frame each 5 s). The sensitivity of this camera was 0.07 °C. For men, the camera was placed 1.0 m diagonally left from the participant and for women, the camera was situated directly facing the genitals at a distance of 1.0 m. Regions of interest (ROIs) have been selected on anatomical bases and following evidence reported in previous published studies^14^. Circular ROIs have been defined on anatomical bases and drawn using FLIR ThermaCAM Researcher Pro version 2.10. For men, a total of 6 ROIs were included: glans of the penis, the penile shaft (two ROIs—distal and proximal portions of the penile shaft), the base of the penis, scrotum, and pubic bone. For women, a total of 7 ROIs were included: glans of the clitoris, cranial and caudal portions of left and right labia majora (four ROIs), left and right sides of mons pubis (two ROI). Temperature, in Celsius degrees, has been extracted from each ROI for each thermogram and used for statistical analyses. For reference, consult Fig. S1.

#### Subjective sexual arousal

Subjective sexual arousal was measured by asking subjects to rate how sexually aroused they felt after each stimulus presentation, using a 9-point Likert scale (1 “*not at all”,* to 9 *“extremely”*).

### Breath trace gas measurements

#### Real-time breath sampling

**Figure 6:**
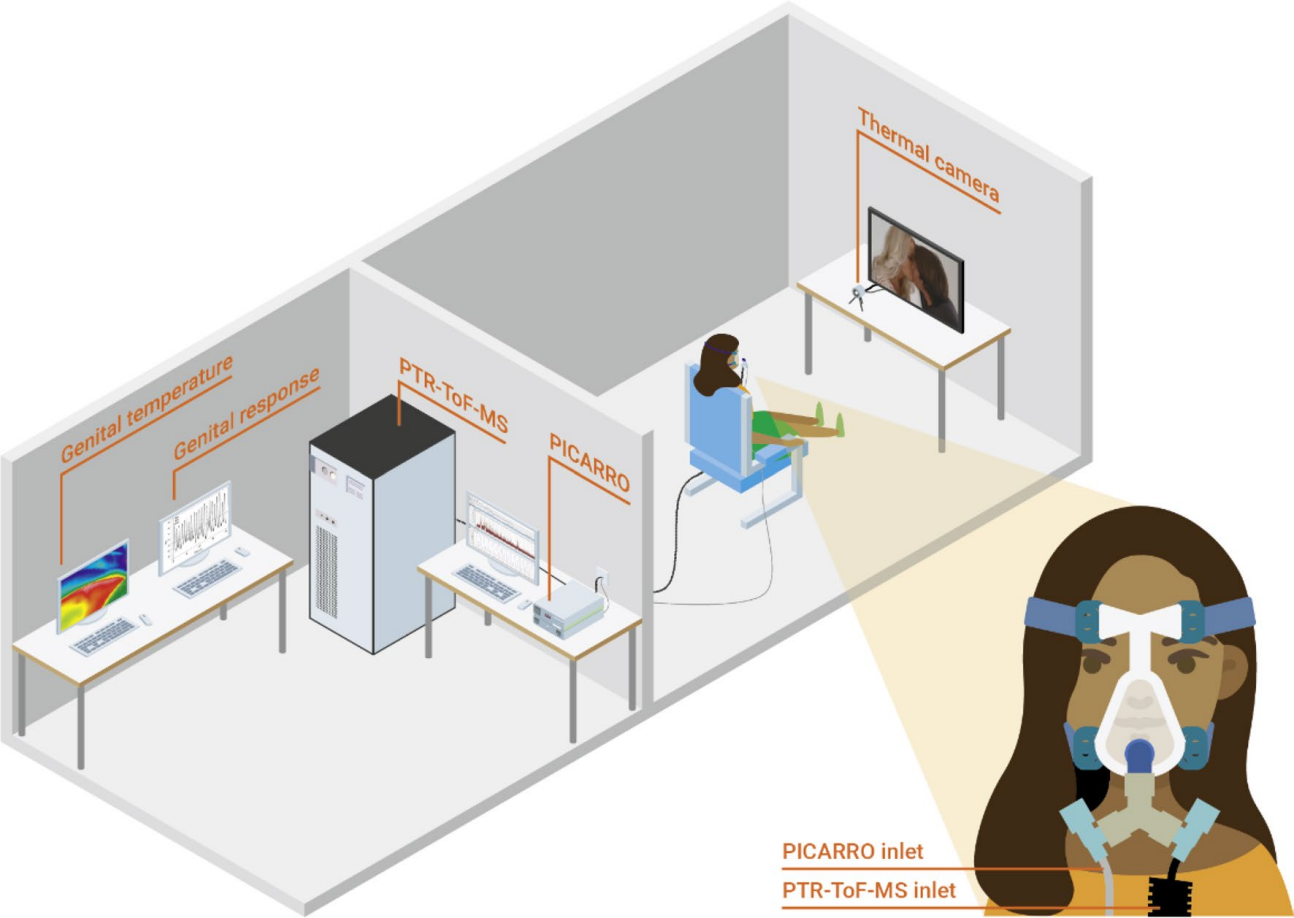
Simplified diagram of the experimental set-up.

For each participant, a sterile face mask (Ultra Mirage™, San Diego, California, USA) attached with a Teflon T-piece was used for continuous breath sampling. A mouthpiece was further connected to each side of the T-piece to allow the side-stream sampling via the Luer connection (Fig. 6) for different trace gas measurement instruments. No breathing resistance was introduced through the mask.

#### VOCs measurement

Breath VOCs measurements were carried out using a proton transfer time of flight mass spectrometer (PTR-ToF-MS-8000, Ionicon Analytik GmbH, Innsbruck, Austria). The working principle of the instrument has been described elsewhere^62^. Briefly, the soft ionization process is based on a proton transfer from hydronium ions (H_3_O^+^) to the sample molecules within the drift tube. Only molecules with proton affinities (PA) higher than water (PA (H_2_O) = 691 kJ mol^−1^) are protonated. Protonated ions are then analyzed in a high resolution reflectron time-of-flight mass spectrometer according to mass-to-charge ratio (m/z). The instrumental settings were as follows. The inlet connected to the side-steam of the mouthpiece (1/16″ silcosteel tubing, 6 m) was heated to 75 °C and the sampling flow was 100 mL/min to optimize the response time for measured compounds^63^. The drift tube pressure was 2.3 mbar, the drift voltage was 600 V, and the drift temperature was 75 °C, resulting in an E/N ratio of 139 Td. The time resolution was 200 ms allowing individual breaths to be resolved, with the m/z monitored up to 500 Da. Data were processed using PTR-MS Viewer v. 3.2.8 software (Ionicon Analytik GmbH, Innsbruck, Austria). Compounds were identified using their protonated m/z and isotopic patterns. The ion abundance of all m/z was measured in counts per second (cps) and to account for possible variations of the reagent ion signals, measured ion intensities were normalized to the H_3_O^+^ counts in combination with the water-cluster ion counts^64^. Only compounds with signal intensities higher than the instrumental background signals were considered for further analysis.

Four-point calibrations were performed using a standard gas mixture containing 14 compounds (Apel-Riemer Environmental inc., Broomfield, USA) under humidified conditions. Compounds that are not presented in the standard, a theoretic calculation method was applied to obtain their mixing ratios^65–67^.

Expiratory and inspiratory phases of each breath were recognized by means of a Matlab based algorithm called “breath tracker” (MATLAB version 7.12.0.635, R2011a). The function of the algorithm has been described previously^63^. Concisely, an endogenous compound that has a higher concentration in expiration compared to the inspiration is used as a tracker mass to differentiate between expired and inspired phases (acetone in this study). Phase resolution determined by means of the algorithm is then applied to all m/z of interest. Only VOCs with mixing ratios in the expired phase higher than the mixing ratios in the inspired phase were considered for further analysis.

#### Other trace gas measurements

Carbon dioxide (CO_2_) and methane (CH_4_) were determined by a cavity ring-down spectrometer (Picarro G2401, Picarro Inc), which drew the side-stream air from the mouth piece via the 1/8″ fluorinated ethylene propylene tubing (~ 6 m). Due to the high loading of CO_2_ in the breath, the breath sample was diluted using the synthetic air (company) before it reached the instrument so that the levels could be maintained within the measurable range.

### Procedures of each experiment

Participants who agreed to take part in the study were asked to answer an online version of a demographic questionnaire, the Beck Depression Inventory (BDI), the State-trait Anxiety inventory (STAI), the International Index of Erectile Function (IIEF) or the Female Sexual Function Index (FSFI), in the privacy of their homes. Upon arrival at the laboratory, participants were instructed on the experimental requirements and, if willing to participate, were asked to sign an informed consent form. At the beginning of each experiment, participants went to a dimly lit private room and were remotely contacted by intercom with instructions on how to place the necessary physiological measurement equipment (male participants were asked to place penile gauges and females inserted the photoplethysmograph involved in a condom). A schematic diagram of the experimental set-up can be found in Fig. 6. Once the participant was ready and comfortable, experiment instructions were displayed on the screen directly opposite the participant. The experiment started with a 3-min neutral video to establish baseline conditions, followed by the three 10-min videos presented in a pseudo-randomized order. The 10-min videos represented the tested conditions (sexual, anxiety and sports). After each film, a 3-min neutral movie was shown allowing the return to baseline state. Particularly after the sexual movie, participants were asked to answer a questionnaire inquiring about subjective sexual arousal, affective responses and self-reported sexual thoughts. Genital response, genital temperature and exhaled breath VOCs were measured continuously over the whole experiment. At the end of the experiment, participants were debriefed, invited to ask further questions or share any concerns about their participation.

### Data normalization

All measured parameters were averaged over 1-min intervals. To reduce inter-individual variations, all parameters were normalized to the corresponding values at the third minute of the first-watched neutral film clip, as this neutral clip reflects the actual baseline conditions without being affected from any other film clip. The third minute was chosen since it is the last minute of the first film shown at the beginning of the experiment and the participants were likely then most settled and used to the experimental set-up. Figures 3, 4, 5 were prepared using the IGOR data processing software by Wavemetrics, version 8.0.

### Statistical analysis

For both male and female group, statistical differences between film for each exhaled trace gas and for each genital parameter were obtained from one-way repeated measures analysis of variance (ANOVA), where Tukey test was used for post-hoc comparisons to determine the origin of difference identified by the ANOVA. A p-value < 0.05 represents significant difference. The analysis was performed with OriginLab (version: 2019b).

### Research involving human participants

The study was approved by the Ethics Committee of the Faculty of Psychology and Educational Sciences of the University of Porto.

### Informed consent

Informed consent was obtained from all volunteers before the study.

## Supplementary Information

Supplementary Information.

## Data Availability

Data are available upon resquest from the authors.
