# Supplementary material for: Breath chemical markers of sexual arousal in humans
Source: Sci Rep. 2022 Apr 15;12:6267. doi: 10.1038/s41598-022-10325-6 (PMC9012850; doi:10.1038/s41598-022-10325-6)
Supplement: Supplementary file 1 — Supplementary Information. [file 41598_2022_10325_MOESM1_ESM.docx]

**Supporting information**

**Breath chemical markers of sexual arousal in humans**

N. Wang^1†^, G. Pugliese^1,4†^, M. Carrito^3^, C. Moura^3^, P. Vasconcelos^3^, N. Cera^3^, M. Li^1^, P. Nobre^3^, J.R. Georgiadis^2^, J.K. Schubert^4^, J. Williams^1,5*^

^1^ Max Planck Institute for Chemistry, Mainz, Germany

^2^ University of Groningen, University Medical Center Groningen, Groningen, Netherlands

^3^ CPUP, Faculty of Psychology and Educational Sciences, University of Porto, Porto, Portugal

^4^ University of Rostock, Rostock, Germany

^5^ Energy, Environment and Water Research Center, The Cyprus Institute, Nicosia, Cyprus.

† Equal contributions

### *Corresponding author: jonathan.williams@mpic.de (ORCID orcid.org/0000-0001-9421-1703)

Figures: S1, S2

Tables: S1, S2


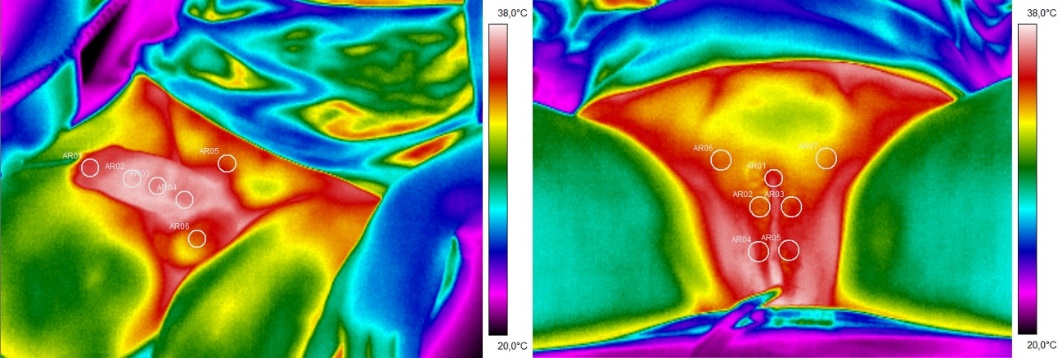


Figure S1. Regions of interest (ROI) of thermographic cameras for men (left) and women (right).


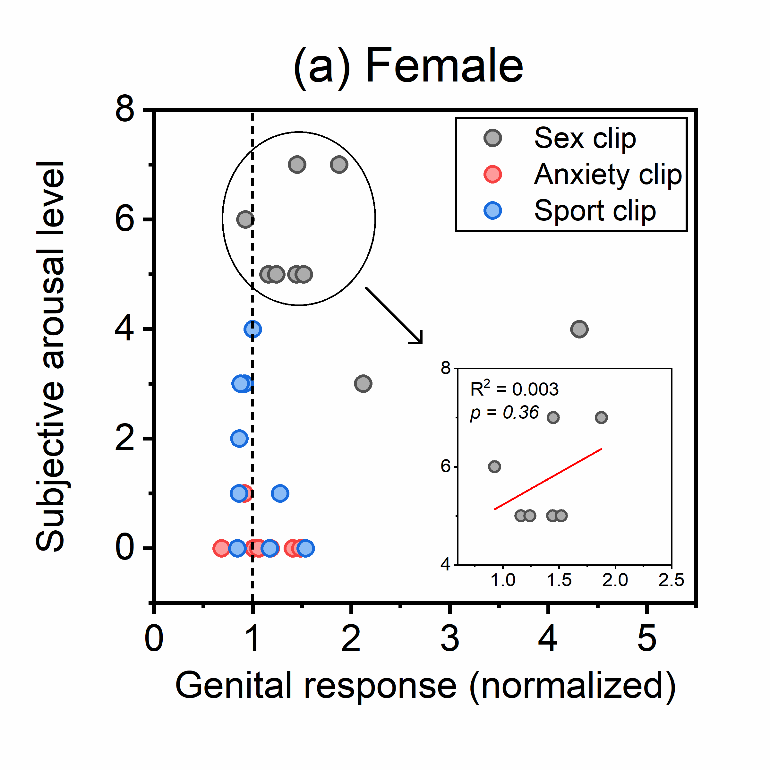

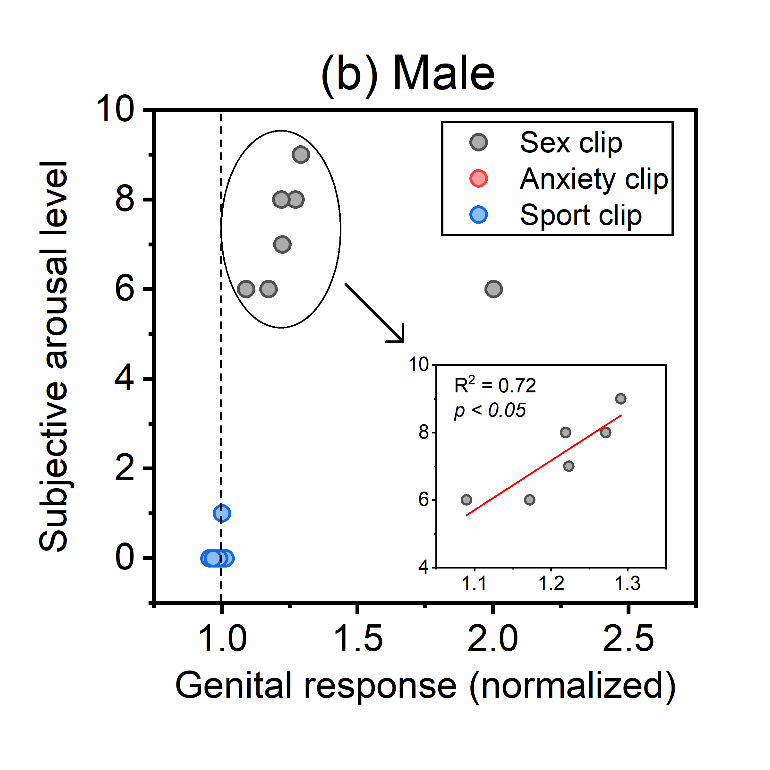


Figure S2. Scatter plots of genital response (female: vaginal pulse amplitude; male: penile circumference) vs. subjective arousal level for (a) female participants (N = 9) and (b) male participants (N = 7). Genital response data were normalized to the third minute of the first neutral clip for each participant. The vertical dash line in each plot represents the value at 1.0 as a reference. The sub-plot in (a) and (b) shows the liner fitting result by excluding the outliers.

Figure S3. Time series of genital response (vaginal pulse amplitude), genital temperature (thermo) and selected VOCs (isoprene, C_7_H_8_O, C_10_H_14_O, and C_8_H_11_NO_2_) for all female participants. Film clips were separated by shaded area in gray which represents the neutral clip. The content of each clip is labelled on the top. The genital response data is not available for participant No.11, No.23 and No.38. The genital temperature data is not available for participant No.18, No.20 and No.26.

Figure S4. Time series of genital response (penile circumference), genital temperature (thermo) and selected VOCs (isoprene, C_6_H_6_O, C_7_H_8_O and C_8_H_7_N) for all male participants except for male participant No.01 who did not have any genital data available. Film clips were separated by shaded area in gray which represents the neutral clip. The content of each clip is labelled on the top. The genital response data is not available for participant No.04, No.07 and No.09. The genital temperature data is not available for participant No.15.

Table S1. Statistic information of mixing ratios (ppbv) of volatile organic compounds (VOCs) measured in participants’ exhalation (N = 24) including mean, median, maximum (max), minimum (min) values and standard deviation (SD)

| **Chemical formula** | (H^+^) m/z | **First Neutral clip (3 min)** | | | | | **Sex clip (10 min)** | | | | | **Anxiety clip (10 min)** | | | | | **Sport clip (10 min)** | | | | |
| --- | --- | --- | --- | --- | --- | --- | --- | --- | --- | --- | --- | --- | --- | --- | --- | --- | --- | --- | --- | --- | --- |
|  |  | mean | median | max | min | SD | mean | median | max | min | SD | mean | median | max | min | SD | mean | median | max | min | SD |
| CH4O^*^ | 33.033 | 514 | 433 | 1528 | 189 | 311 | 475 | 416 | 1488 | 184 | 286 | 470 | 395 | 1293 | 178 | 270 | 470 | 409 | 1299 | 176 | 255 |
| C2H3N^*^ | 42.034 | 3.64 | 1.26 | 39.5 | 0.510 | 7.95 | 3.40 | 1.20 | 37.92 | 0.555 | 7.28 | 3.50 | 1.19 | 39.1 | 0.51 | 7.69 | 3.46 | 1.23 | 37.4 | 0.538 | 7.38 |
| C2H4O^*^ | 45.033 | 33.5 | 23.0 | 132 | 3.045 | 29.8 | 32.1 | 23.7 | 124.2 | 3.14 | 27.4 | 30.6 | 20.9 | 99.2 | 3.13 | 26.0 | 29.2 | 24.3 | 83.2 | 2.92 | 21.4 |
| C3H6O^*^ | 59.049 | 293 | 282 | 561 | 113 | 111 | 295 | 274 | 538 | 110 | 110 | 297 | 271 | 558 | 126 | 107 | 300 | 282 | 550 | 134 | 106 |
| C2H4O2^**^ | 61.028 | 7.83 | 7.21 | 18.2 | 3.30 | 2.82 | 7.64 | 7.33 | 16.15 | 3.06 | 2.30 | 7.16 | 6.69 | 18.26 | 3.14 | 2.54 | 7.24 | 6.63 | 16.9 | 3.06 | 2.42 |
| C3H8O^**^ | 61.065 | 0.857 | 0.736 | 2.51 | 0.184 | 0.542 | 0.842 | 0.676 | 2.35 | 0.168 | 0.509 | 0.848 | 0.692 | 2.335 | 0.164 | 0.521 | 0.858 | 0.747 | 2.50 | 0.191 | 0.519 |
| C2H6S^*^ | 63.026 | 4.17 | 3.41 | 10.5 | 1.22 | 2.43 | 4.09 | 3.07 | 10.15 | 1.34 | 2.44 | 4.09 | 3.22 | 10.40 | 1.33 | 2.40 | 4.22 | 3.25 | 10.7 | 1.23 | 2.58 |
| C5H8^*^ | 69.070 | 115 | 114 | 309 | 29.9 | 54.2 | 115 | 109 | 384 | 28.6 | 57.5 | 120 | 115 | 296 | 37.6 | 56.1 | 128 | 113 | 349 | 41.5 | 64.2 |
| C3H8O2^**^ | 77.060 | 0.530 | 0.461 | 1.59 | 0.094 | 0.309 | 0.499 | 0.446 | 1.47 | 0.088 | 0.266 | 0.515 | 0.468 | 1.46 | 0.097 | 0.301 | 0.519 | 0.473 | 1.51 | 0.089 | 0.294 |
| C5H10O^**^ | 87.080 | 0.484 | 0.299 | 2.74 | 0.205 | 0.492 | 0.434 | 0.285 | 2.77 | 0.164 | 0.439 | 0.424 | 0.295 | 2.10 | 0.172 | 0.359 | 0.407 | 0.293 | 1.89 | 0.173 | 0.303 |
| C4H10S^**^ | 91.058 | 0.765 | 0.706 | 1.45 | 0.441 | 0.242 | 0.760 | 0.697 | 1.45 | 0.467 | 0.226 | 0.761 | 0.706 | 1.54 | 0.403 | 0.240 | 0.758 | 0.693 | 1.56 | 0.405 | 0.254 |
| C6H6O^**^ | 95.049 | 0.571 | 0.393 | 2.78 | 0.200 | 0.530 | 0.639 | 0.355 | 3.82 | 0.215 | 0.700 | 0.550 | 0.344 | 3.75 | 0.199 | 0.673 | 0.558 | 0.349 | 3.29 | 0.180 | 0.591 |
| C7H8O^**^ | 109.065 | 0.087 | 0.070 | 0.438 | 0.039 | 0.071 | 0.175 | 0.064 | 4.25 | 0.030 | 0.535 | 0.099 | 0.065 | 1.68 | 0.026 | 0.175 | 0.142 | 0.067 | 4.76 | 0.032 | 0.470 |
| C8H7N^**^ | 118.065 | 0.063 | 0.036 | 0.344 | 0.012 | 0.071 | 0.071 | 0.029 | 0.512 | 0.011 | 0.103 | 0.064 | 0.030 | 0.570 | 0.005 | 0.103 | 0.058 | 0.029 | 0.466 | 0.004 | 0.086 |
| C8H11N^**^ | 122.096 | 0.024 | 0.024 | 0.040 | 0.013 | 0.006 | 0.024 | 0.024 | 0.049 | 0.010 | 0.006 | 0.024 | 0.023 | 0.040 | 0.008 | 0.007 | 0.024 | 0.024 | 0.04 | 0.007 | 0.006 |
| C8H16O^**^ | 129.127 | 0.823 | 0.599 | 3.53 | 0.134 | 0.765 | 0.617 | 0.410 | 3.49 | 0.106 | 0.634 | 0.623 | 0.460 | 2.80 | 0.118 | 0.568 | 0.580 | 0.427 | 2.42 | 0.109 | 0.467 |
| C10H16^*^ | 137.132 | 3.06 | 2.83 | 5.98 | 1.90 | 0.81 | 3.06 | 2.81 | 7.36 | 1.92 | 0.94 | 2.94 | 2.76 | 6.19 | 1.87 | 0.76 | 3.00 | 2.79 | 6.92 | 1.84 | 0.85 |
| C8H16O2^**^ | 145.122 | 0.324 | 0.205 | 1.51 | 0.131 | 0.328 | 0.302 | 0.209 | 1.51 | 0.117 | 0.268 | 0.295 | 0.207 | 1.58 | 0.122 | 0.275 | 0.315 | 0.210 | 1.63 | 0.106 | 0.317 |
| C10H14O^**^ | 151.110 | 0.162 | 0.125 | 1.10 | 0.076 | 0.169 | 0.476 | 0.117 | 13.8 | 0.078 | 1.761 | 0.233 | 0.119 | 5.18 | 0.078 | 0.567 | 0.352 | 0.117 | 15.2 | 0.082 | 1.507 |
| C8H11NO2^**^ | 154.086 | 0.030 | 0.027 | 0.073 | 0.012 | 0.012 | 0.032 | 0.029 | 0.138 | 0.008 | 0.018 | 0.029 | 0.027 | 0.081 | 0.003 | 0.012 | 0.030 | 0.026 | 0.156 | 0.009 | 0.017 |
| C10H8O3^**^ | 177.055 | 0.054 | 0.050 | 0.116 | 0.022 | 0.020 | 0.056 | 0.049 | 0.183 | 0.023 | 0.026 | 0.053 | 0.050 | 0.128 | 0.021 | 0.022 | 0.055 | 0.048 | 0.143 | 0.023 | 0.022 |

* VOCs calibrated with standard gas mixture (CH4O: methanol; C2H3N: acetonitrile; C2H4O: acetaldehyde; C3H6O: acetone; C2H6S: dimethyl sulfide; C5H8: isoprene; C10H16: α-pinene).

** Potential chemical assignment: C2H4O2 (acetic acid), C3H8O (propanol), C3H8O2 (unknown), C5H10O (pentanone/pentanol), C4H10S (diethyl sulphide), C6H6O (phenol), C7H8O (cresol), C8H7N (indole), C8H11N (Phenethylamine), C8H16O (octenol/octanal/octanone), C8H16O2 (octanoic acid/hexyl acetate), C10H14O (2-ethyl-4,5-dimethylphenol), C8H11NO2 (dopamine), C10H8O3 (hymecromone).

Table S2. F-statistics results from One Way Repeated Measures ANOVA and P values from Tukey post-hoc test for pairwise comparison among film clips of different VOCs for male and female participants (numbers in bold representing the difference between two clips is significant where p < 0.05, with positive difference in red and negative difference in blue).

| **Male (*N=12*)** | | | | |  | **Female (*N=12*)** | | | | | |
| --- | --- | --- | --- | --- | --- | --- | --- | --- | --- | --- | --- |
|  | F(df_clip_, df_error_) = F_value_, p-value | Sex vs. Anxiety | Sex vs. Sport | Anxiety vs. Sport | F(df_clip_, df_error_) = F_value_, p-value | | Sex vs. Anxiety | Sex vs. Sport | Anxiety vs. Sport | |  |
| Genital response^*^ | (1,79.4) = 67.62, p <0.05 | **<0.0001** | **<0.0001** | 0.9991 | (1.2,105.8) = 46.88, p <0.05 | | **<0.0001** | **<0.0001** | 0.8482 |  |  |
| Temperature^*^ | (1.3, 131.3) = 103.03, p<0.05 | **<0.0001** | **<0.0001** | **<0.0001** | (1.7, 153.3) = 7.65, p<0.05 | | 0.3286 | **0.0004** | **0.0412** |  |  |
| Subjective arousal^*^ | (1.1, 9.77) = 393.31, p<0.05 | **<0.0001** | **<0.0001** | 1.0000 | (2, 22) = 82.41, p<0.05 | | **<0.0001** | **<0.0001** | **0.0284** |  |  |
| CO2 | (1.8, 213.4) = 13.96, p<0.05 | **0.0002** | **<0.0001** | 0.6306 | (2, 238) = 8.49, p<0.05 | | **0.0017** | **0.0009** | 0.9861 |  |  |
| CH4 | (1.6, 192.9) = 9.26, p<0.05 | **<0.0001** | 0.2344 | **0.0244** | (1.7, 207.9) = 2.69, p>0.05 | | 0.1199 | 0.9980 | 0.1054 |  |  |
| Acetonitrile | (1.9, 231.6) = 8.93, p<0.05 | 0.5032 | **0.0092** | **0.0002** | (2, 238) = 2.66, p>0.05 | | 0.1829 | 0.0786 | 0.9146 |  |  |
| Acetaldehyde | (1.7, 206.91) = 5.33, p<0.05 | **0.0149** | 1.0000 | **0.0133** | (1.5, 183.6) = 7.79, p<0.05 | | **0.0013** | **0.0037** | 0.9483 |  |  |
| C2H4O2 | (1.8, 216.6) = 14.44, p<0.05 | **<0.0001** | **0.0002** | 0.5978 | (1.4, 171.6) = 2.2, p>0.05 | | 0.1598 | 0.1730 | 0.9991 |  |  |
| C3H8O | (2, 238) = 9.45, p<0.05 | 0.6406 | **0.0040** | **0.0001** | (1.8, 215.1) = 2.03, p>0.05 | | 0.2186 | 0.9886 | 0.1669 |  |  |
| DMS | (2, 238) = 13.10, p<0.05 | 0.6467 | **0.0003** | **<0.0001** | (2, 238) = 2.91, p>0.05 | | 0.0504 | 0.2308 | 0.7550 |  |  |
| Isoprene | (1.74, 207.4) = 12.55, p<0.05 | 0.9993 | **<0.0001** | **<0.0001** | (2, 238) = 15.58, p<0.05 | | **<0.0001** | **<0.0001** | 0.6566 |  |  |
| C3H8O2 | (2, 238) = 10.39, p<0.05 | 0.1676 | **0.0194** | **<0.0001** | (1.90, 225.9) = 5.63, p<0.05 | | **0.0038** | 0.6414 | 0.0509 |  |  |
| C5H10O | (1.9, 223.9) = 2.10, p>0.05 | 0.1581 | 0.9903 | 0.2037 | (2, 238) = 8.40, p<0.05 | | **0.0008** | 0.9524 | **0.0023** |  |  |
| C4H10S | (1.9, 221.4) = 0.16, p>0.05 | 0.9870 | 0.9149 | 0.8434 | (1.8, 215.9) = 5.8, p<0.05 | | 0.8794 | **0.0050** | **0.0215** |  |  |
| C6H6O | (1.35, 160.64) = 9.12, p<0.05 | **0.0002** | **0.0036** | 0.7306 | (1.6, 194.6) = 1.28, p>0.05 | | 0.2581 | 0.8554 | 0.5504 |  |  |
| C7H8O | (1.85, 219.97) = 0.57, p>0.05 | 0.6661 | 0.9925 | 0.5923 | (2, 238) = 4.62, p<0.05 | | **0.0076** | 0.4012 | 0.1932 |  |  |
| C8H7N | (1.57, 187.29) = 4.52, p<0.05 | **0.0497** | **0.0156** | 0.9027 | (1.4, 172.6) = 0.38, p>0.05 | | 0.7298 | 0.9999 | 0.7356 |  |  |
| C8H11N | (1.89, 224.55) = 1.88, p>0.05 | 0.5023 | 0.1316 | 0.6954 | (2, 238) = 0.91, p>0.05 | | 0.4020 | 0.5828 | 0.9520 |  |  |
| Monoterpenes | (2, 238) = 10.96, p<0.05 | **<0.0001** | 0.3781 | **0.0042** | (2, 238) = 8.48, p<0.05 | | **0.0002** | 0.0648 | 0.1527 |  |  |
| C8H16O2 | (1.67, 199.04) = 4.92, p<0.05 | **0.0054** | 0.2353 | 0.2887 | (1.5, 177.8) = 0.62, p>0.05 | | 0.9384 | 0.7360 | 0.5234 |  |  |
| C10H14O | (2, 238) = 2.42, p>0.05 | 0.5190 | 0.0728 | 0.5095 | (1.9, 225) = 4.18, p<0.05 | | **0.0117** | 0.2829 | 0.3595 |  |  |
| C8H11NO2 | (2, 238) = 0.87, p>0.05 | 0.9995 | 0.4989 | 0.4799 | (2, 238) = 6.87, p<0.05 | | **0.0008** | 0.2179 | 0.1073 |  |  |
| C10H8O3 | (2, 238) = 3.83, p<0.05 | 0.2020 | 0.5607 | **0.0180** | (1.8, 210.1) = 3.68, p<0.05 | | 0.0963 | **0.0306** | 0.8855 |  |  |

* having different group size: genital response (male: 8; female: 9), temperature (male: 10; female: 9), subjective arousal (male: 10, female: 12)
